# A Maternal–Offspring Coadaptation Theory for the Evolution of Genomic Imprinting

**DOI:** 10.1371/journal.pbio.0040380

**Published:** 2006-11-14

**Authors:** Jason B Wolf, Reinmar Hager

**Affiliations:** Faculty of Life Sciences, The University of Manchester, Manchester, United Kingdom; University of Edinburgh, United Kingdom

## Abstract

Imprinted genes are expressed either from the maternally or paternally inherited copy only, and they play a key role in regulating complex biological processes, including offspring development and mother–offspring interactions. There are several competing theories attempting to explain the evolutionary origin of this monoallelic pattern of gene expression, but a prevailing view has emerged that holds that genomic imprinting is a consequence of conflict between maternal and paternal gene copies over maternal investment. However, many imprinting patterns and the apparent overabundance of maternally expressed genes remain unexplained and may be incompatible with current theory. Here we demonstrate that sole expression of maternal gene copies is favored by natural selection because it increases the adaptive integration of offspring and maternal genomes, leading to higher offspring fitness. This novel coadaptation theory for the evolution of genomic imprinting is consistent with results of recent studies on epigenetic effects, and it provides a testable hypothesis for the origin of previously unexplained major imprinting patterns across different taxa. In conjunction with existing hypotheses, our results suggest that imprinting may have evolved due to different selective pressures at different loci.

## Abstract 2

This theoretical paper demonstrates that maternal-offspring coadaptation (the selection for gene combinations of parents and offspring) could select for genomic imprinting of the offspring trait.

## Introduction

The term “genomic imprinting” refers to the phenomenon of parent-of-origin–dependent gene expression, whereby at a given locus, either the maternally or the paternally inherited copy is expressed while the other ‘imprinted' copy remains silent [1]. Since the discovery of genomic imprinting in the mid 1980s [2], there has been considerable interest among evolutionary biologists to elucidate both the mechanisms and the selective forces behind the evolutionary origin of imprinting [3–6]. The occurrence of this pattern of “monoallelic” gene expression presents an evolutionary paradox, because it negates the advantage of diploidy by exposing recessive deleterious mutations and, thereby, incurs a fitness cost [7,8]. Thus, a critical step in understanding the evolution of imprinting is to recognize why selection may favor imprinting of certain loci.

Currently favored views on the evolution of genomic imprinting maintain that uniparental expression of genes is largely the result of evolutionary conflict between males and females over the level of maternal investment [6,9,10]. Recent theory also suggests that imprinting may stem from intralocus sexual conflict [11]. The former hypothesis posits that imprinting may generally be expected to evolve whenever a locus incurs asymmetrical fitness consequences for paternally versus maternally derived alleles [6,9,10]. According to this kinship theory (or conflict hypothesis), selection favors the sole expression of paternally derived alleles that increase maternal resource allocation to offspring while the maternally inherited copy remains silent (e.g., *Igf2* [9] and *Peg3* [12]). Paternally derived growth enhancers gain fitness benefits from increased maternal investment but do not suffer the “fitness cost” of reduced residual maternal reproductive success due to the effects of multiple paternity, which results in offspring being more closely related via their maternally inherited genes than they are through their paternal genes. Similarly, growth inhibitors are predicted to be expressed from the maternally derived allele only [9,13–16]. By contrast, the intralocus sexual conflict hypothesis states that genomic imprinting extenuates the conflict caused by sex-specific selection favoring different alleles in males and females, for example, when sexual selection results in directional selection in only one sex [11].

Selection, however, often favors the coadaptation of complementary maternal and offspring traits that positively affect offspring development and fitness, suggesting that specific maternal–offspring interactions may reflect adaptive integration of coadapted maternal and offspring traits rather than conflict [17]. This view is supported by studies in a diversity of taxa demonstrating an adaptive genetic correlation between maternal and offspring traits, such as provisioning and offspring solicitation [18–20]. For example, cross-fostering experiments in great tits *(Parus major)* revealed that the level of offspring begging behavior and maternal response was correlated [18], which was also found in insects with parental care [19,20]. Similarly, in mammals, young mice received more provisioning from mothers of their own maternal strain [21]. Genetic coadaptation has also been found between offspring performance and maternal choice of oviposition site (e.g., in the herbivorous fly, Liriomyza sativae) [22]. Furthermore, analyses of selection on human birth weight [23] have revealed that offspring with intermediate birth weights have highest fitness, showing that natural selection does not favor ever increasing investment of maternal resources. Theoretical work has demonstrated that this situation may lead to selection favoring coadaptation of maternal and offspring traits to achieve the optimal birth weight [24].

The best documented cases of maternal–offspring interactions and their effects on early development are found in mammals, in which placental growth is affected by genomic imprinting [25–27]. Indeed, all known imprinted autosomal genes that are exclusively imprinted in the placenta (and not in other tissues as well) of mice are expressed from the maternal allele only [27,28]. Here we show that imprinting with maternal expression is most likely to evolve when selection favors coadapted maternal and offspring traits, for example during mammalian development in utero [29,30]. In addition, by providing a mechanism for the evolution of maternal expression, our model yields a potential explanation for the predicted large number and relative overabundance of maternally expressed genes as suggested by a recent analysis of the murine genome [31]. To demonstrate the generality of this result, we consider the two possible modes through which genetic coadaptation between mothers and offspring can be achieved: pleiotropy and linkage disequilibrium (see [24]). Pleiotropy can generate coadaptation, because the same locus affects both the maternal and offspring traits involved in the interaction, and thereby may facilitate the adaptive fit between them. We examine this case using in a single-locus model in which both maternal and offspring traits involved in the coadaptation are affected by the same locus. Linkage disequilibrium generates coadaptation because it allows for coadapted alleles to be associated in the genome, such that the alleles expressed by offspring will complement those expressed by their mothers. This scenario is modeled using a two-locus model. In both models, we assume that selection favors coadaptation such that offspring fitness is determined by the combination of its own genotype and that of its mother [24,30,32]. Although we develop our model in terms of maternal–offspring interactions, the framework applies equally well to coadapted paternal-offspring interactions when fathers are the primary caregivers.

### Description of the Model

#### Single-locus model.

In the single-locus “pleiotropy model,” we assume that an autosomal locus *(A)* with two alleles, *A*_1_ and *A*_2_ *,* with frequencies *p* and *q,* respectively, affects the expression of both a maternal and an offspring trait. We assume a diploid sexual population with random mating that is sufficiently large to ignore effects of inbreeding and drift. Because we wish to explore the effects of genomic imprinting, we distinguish the two heterozygotes that differ with respect to the parent-of-origin of their two alleles (*A*_1_ *A*_2_ versus *A*_2_ *A*_1_: alleles in genotypes are given as maternal/paternal) [33]. We assume a simple additive model for trait expression, in which the *A* locus has an additive effect on both the maternal and offspring trait, with the phenotypic values of these traits designated by *m_i_* and *o_i_,* respectively, with subscripts *i* equal to 1 = *A*_1_ *A*_1_ *,* 2 = *A*_1_ *A*_2_ *,* 3 = *A*_2_ *A*_1_ *,* and 4 = *A*_2_ *A*_2_. The additive effect of the locus on the maternal trait is designated *a_m_* (where genotypes are designated such that *a_m_* > 0), and the phenotypic values of the maternal trait are defined as: *m*_1_ = +*a_m_, m*_2_ = *m*_3_ = 0, and *m*_4_ = –*a_m_* (both types of heterozygous mothers are assumed to be equivalent). We assume that there is no imprinting of the maternal trait, because we found that imprinting of the maternal trait is neither favored nor disfavored by selection under our assumed model (JB Wolf, R Hager, unpublished data). In cases where the locus shows an imprinted effect on the offspring traits, we assume that without selection directly favoring imprinting of the maternal trait, the locus would evolve to a nonimprinted state during adulthood. This assumption is reasonable given that imprinting can be very tissue- and ontogenetic stage–specific [34], suggesting that control of imprinted expression can be very flexible and evolve to show specific patterns.

The additive effect on the offspring trait is designated *a_o_* (where genotypes are designated such that *a_o_* > 0). We assume that the effect of the locus on the offspring trait is modified by the presence of genomic imprinting with maternal expression. The parameter *I* denotes the degree of imprinting such that when *I* = 0, there is no imprinting and when *I* = 1, there is complete inactivation or silencing of the paternal allele (equivalent to “paternal imprinting” or “maternal expression”). A negative value of *I* would indicate maternal silencing or expression of the paternal allele, but we restrict our discussion of imprinting to positive values of *I* (i.e., silencing of the paternal allele) because coadaptation does not favor negative values of *I* (which would result in a mismatch of coadapted maternal and offspring traits, and therefore, imprinting would disrupt the genetic integration of these traits). We further assume that the effect of the hemizygous expression of an allele is equivalent to its effect in a homozygote such that the phenotypic values of the four offspring genotypes are *o*_1_ = +*a_o_, o*_2_ = +*Ia_o_, o*_3_ = –*Ia_o_,* and *o*_4_ = –*a_o_*. We make no assumption about the control of imprinting, and our model is compatible with assuming either *cis* control of imprinting, whereby a locus determines it own level of imprinting, or *trans* control, in which a different locus controls imprinting at locus *A* [11]. In the latter case, it can be shown that the parameter *I* is equivalent to the frequency of an allele at a second (*trans* acting) locus that leads to imprinting at locus *A* (JB Wolf, R Hager, unpublished data).

We develop a simple selection model in which offspring fitness is determined by the interaction between its own *A* locus genotype and the *A* locus genotype of its mother [30,35,36], such that the effect of the maternal trait (genotype) on offspring fitness depends on the offspring's own trait (genotype). Offspring fitness, expressed as a function of offspring *A* locus genotypes and maternal *A* locus genotypes, is designated *w_ij_,* with subscript *i* indicating the maternal genotype, *j* indicating the offspring genotype, and with values of the subscripts as given above for the phenotypic values. Formally, offspring fitness is defined by *w_ij_* = *μ* + *m_i_o_j_s,* where *s* denotes the strength of the interaction effect between offspring and maternal genotype on offspring fitness (the assumption of selection favoring coadaptation implies *s* > 0) and *μ* is mean fitness independent of the effects of the maternal–offspring interaction (we assume that fitness effects are scaled so that fitness is non-negative, i.e., *w_ij_* ≥ 0). This scenario can be thought of as the maternal trait creating a “maternally provided environment” in which offspring develop, and the offspring trait determining the adaptation or response to this maternally provided environment. This fitness model is equivalent to assuming that offspring phenotype is determined by the interaction of maternal and offspring genotypes (e.g., during placental development [25]), whereby *a_m_* and *a_o_* are the relative effects of the maternal and offspring genotypes on offspring phenotype, and *s* is the strength of selection on the latter. This assumption is supported by empirical studies of maternal effects on offspring fitness. For example, embryo transfer experiments in mice have shown that maternal uterine effects on offspring growth depend on offspring genotype [29]. Similar results were found in studies on postnatal maternal effects in mice [36–39], maternal provisioning in insects and birds [18–20], and oviposition site choice and offspring performance in insects [22].

The frequencies of maternal–offspring genotype combinations under the model assumptions are designated *F_ij_,* with subscripts following those of *w_ij_*. These maternal–offspring frequencies are derived and given in the Materials and Methods section. Population mean fitness (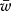 ) is calculated from the maternal–offspring genotype combination frequencies and their fitness (i.e., 
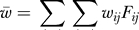
 ) and can be expressed as a function of allele frequencies, the strength of the effects of the *A* locus on the maternal and offspring traits, the degree of imprinting, and the strength of selection:

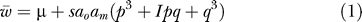



#### Two-locus model.

The two-locus “linkage model” is identical to the one-locus model, except we now assume that one locus, *M,* with two alleles, *M*_1_ and *M*_2_ *,* determines the maternal trait that affects offspring fitness, whereas a second autosomal locus, *O,* with two alleles, *O*_1_ and *O*_2_ *,* determines the offspring trait that directly affects offspring fitness. We use the same structure as in the single locus model, but substitute alleles *A*_1_ and *A*_2_ with *M*_1_ and *M*_2_ *,* respectively, for the maternal trait and *O*_1_ and *O*_2_ for the offspring trait. For the sake of clarity, we focus in the following presentation only on the differences to the one-locus model.

The frequencies of the four alleles at the two loci are designated *p, q, x,* and *y* for the *M*_1_ *, M*_2_ *, O*_1_ *,* and *O*_2_ alleles, respectively. Offspring fitness is determined by the combination of the individual's *O* locus and its mother's *M* locus genotype with fitness values *(w_ij_)*. Likewise, the frequencies of maternal–offspring genotype combinations *(F_ij_)* are given for the maternal *M* locus and offspring *O* locus. These frequencies are derived and presented in the Materials and Methods section. Population mean fitness, calculated as above, is now determined by the fitness values and frequencies of the maternal *M*-locus–offspring *O*-locus combinations, and is given by

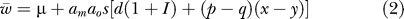



For both models, we used the genetic covariance between the maternal and offspring traits [cov(*m*,*o*)] to quantify the adaptive genetic integration of traits expressed in mothers and their offspring [24]. This covariance (generally referred to as the “direct-maternal” covariance, [24,40]) is calculated as: 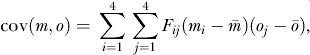 , where 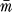 and 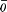 are the means of the maternal and offspring traits, respectively. The separate covariance expressions for the two models are presented below in the Results section.

## Results

The key question of whether the assumed model of selection favors the evolution of genomic imprinting can be examined by analyzing the effect of the level of imprinting on population mean fitness *(* 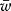 *)*. In the single-locus model, partial differentiation of population mean fitness (Equation 1) with respect to the level of imprinting yields a simple expression for the effect of imprinting on population mean fitness

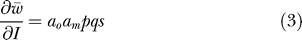



This equation demonstrates that imprinting is favored by selection whenever there is genetic variation at a locus affecting the maternal and offspring traits involved in the maternal–offspring interaction. The strength of selection favoring imprinting will depend on the strength of the effect that the locus has on the offspring *(a_o_)* and maternal traits *(a_m_)* and the intensity of selection *(s)* acting on maternal–offspring genotype combinations. Thus, Equation 3 demonstrates the simple result that the evolution of imprinting is favored whenever there is genetic variation affecting coadapted maternal and offspring traits. Although our assumed model of selection is expected to lead to the erosion of genetic variation as a means of achieving coadaptation, genetic variation for maternal and offspring traits appears ubiquitous in natural populations [41–43], as does variation for traits under natural selection in general [44]. For example, patterns of genetic variation that are consistent with our model have been found in many systems in which imprinting plays an important role [24,30], such as prenatal maternal-fetal interactions [29]. Thus, it seems reasonable to assume that variation for the maternal–offspring interaction exists in most populations and can play a role in the evolution of imprinting.

Genomic imprinting increases population mean fitness because it increases the adaptive genetic integration of maternal and offspring traits. This can be seen by examining the covariance between the maternal and offspring traits [cov(*m*,*o*)]

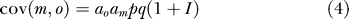
which shows that complete imprinting (*I* = 1) is expected to double the maternal–offspring genetic covariance. In the absence of imprinting, the covariance has the value 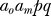 , which also appears in Equation 3 where it is multiplied by the strength of selection *(s)* to calculate the effect of imprinting on population mean fitness. This confirms that the effect of imprinting on population mean fitness is due to its effect on increasing the maternal–offspring genetic covariance.

For the two-locus model, the effect of imprinting on population mean fitness is again given by the partial derivative of population mean fitness with respect to the level of imprinting, which yields

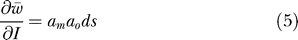



This expression shows that imprinting is favored whenever there is linkage disequilibrium between the pair of loci affecting the maternal and offspring traits. Linkage disequilibrium will always be nonzero when selection favors coadaptation, because selection increases the frequency of maternal and offspring genotype combinations that result in high offspring fitness [24,30,32]. Formally, this can be demonstrated by finding the partial derivative of population mean fitness with respect to the level of disequilibrium

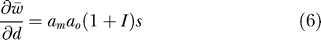
which confirms that the assumed pattern of selection is expected to build linkage disequilibrium between loci. In other words, selection builds linkage disequilibrium because it leads to the integration of maternal and offspring traits [24,30,32]. Equation 6 also shows that imprinting facilitates the process of coadaptation by leading to stronger selection favoring linkage disequilibrium between maternal and offspring loci, thereby enhancing the level of integration achieved.

In both models, imprinting has the same effect on maternal–offspring coadaptation: in the single-locus model, imprinting has the potential to double the genetic covariance between maternal and offspring traits, and in the two-locus model, imprinting doubles the contribution of linkage disequilibrium to the genetic covariance

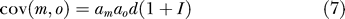



Thus, again, imprinting affects population mean fitness via its effects on the maternal–offspring genetic covariance.

## Discussion

The key result of our model is that natural selection favors the evolution of genomic imprinting, because it increases offspring fitness by enhancing the genetic integration of coadapted offspring and maternal traits. Our model provides a number of testable predictions, which, in conjunction with existing hypotheses, may yield a better understanding of the functional basis for the evolutionary origin of imprinting across the genome. First, central to our model is the prediction that in systems where selection favors coadaptation, the loci involved in the intimate maternal–offspring interaction are more likely to show patterns of imprinting with maternal expression. The predominance of maternally expressed genes in both placental [28] and early seed development [45] may thus be explained by our model. In the case of the placenta, the kinship hypothesis, the intralocus conflict hypothesis, and our coadaptation model predict imprinting to occur at different loci and/or at different stages of development, but only the coadaptation hypothesis makes the explicit prediction of the predominance of maternally expressed genes. Thus, the coadaptation hypothesis may explain the abundance of maternally expressed genes, especially at loci affecting traits that are vital for the development of a functional placenta, such as those expressed early in development or at the interface of the maternal–fetal interaction (e.g., at the boundary of the maternal and embryonic contributions to the placenta). By contrast, the kinship hypothesis may explain the occurrence of imprinting at loci that govern resource transfer between embryo and mother (e.g., *Igf2* and *Igf2r*) [9], where an opportunity for conflict over optimal levels of maternal investment exists (with expression of maternally or paternally inherited alleles, depending on the effect of the locus). Finally, the intralocus sexual conflict hypothesis may explain the occurrence of imprinting at loci with alleles under differential directional selection in males and females, but therefore may not make any specific predictions regarding the pattern of imprinting in the placenta if differential selection does not occur at such an early stage of development.

Second, we expect the incidence of imprinting at loci affecting traits involved in maternal–offspring interactions to be higher in taxa in which such interactions have a greater impact on offspring fitness. Like the intralocus sexual conflict model, our results also suggest that imprinting may occur in systems that previously have not been the focus of research on genomic imprinting because they do not offer the opportunity for conflict over maternal investment, but in which coadapted maternal–offspring traits have potentially large fitness effects (e.g., systems where selection favors coadaptation of oviposition site preference and offspring performance, such as in many phytophagous insects [46]). Our model focuses on maternal–offspring interactions, but it would be interesting to investigate whether our predictions hold for systems in which the father is the primary caregiver, such as in several fish or arthropod species [47,48]. Given that the coadaptation of paternal and offspring traits has fitness consequences for offspring, we would expect expression of the paternal allele.

Finally, although we offer an alternate theory for the evolution of genomic imprinting, we stress that the diversity of imprinting patterns and associated phenotypic effects found across different genes suggests the possibility that imprinting may have evolved for different reasons in different taxa and loci [11,49]. This applies, in particular, to maternal–offspring interactions, in which some of the underlying genes may be involved in conflict over maternal provisioning while others might be expressed for their role in maternal–offspring coadaptation, either at different stages in development or in different tissues.

At present, most evidence in support of or against theories of imprinting comes from studies on phenotypic effects identified by gene targeting [6]. However, studying the effects of mutated genes may provide only limited insight into the evolutionary causes underlying imprinting at specific loci, because these neither necessarily reflect the evolutionarily important functions of the genes in question nor naturally occurring patterns of allelic variation at such loci. For example, mutating a gene that plays an important role in placental development might result in retarded (or perhaps enhanced) offspring growth, but the evolutionarily important effect of the gene may be proper development of a functional placenta rather than growth promoting or inhibition, per se. Thus, such a gene could have evolved maternal expression due to coadaptation of alleles that function together for proper placental development, whereas effects on growth in knock-out mutants may reflect pleiotropic outcomes of severe genetic perturbations. Experimental and comparative analyses designed to directly test alternative theories for the evolutionary origin of imprinting are required to elucidate the diversity of imprinting patterns at both the genomic and taxonomic level [50]. Such analyses might compare different species that show different patterns of maternal–offspring interaction and coadaptation to test whether loci involved in coadaptation or conflict show predicted patterns of imprinting. Likewise, analyses within species might examine the effects of allelic variation at loci showing maternal versus paternal expression to test whether such loci show patterns consistent with coadaptation. For such a test, one might examine whether the effects of imprinted loci with maternal expression depend on the genotype of the mother and whether the resulting pattern is consistent with selection for coadaptation. Thus, by offering a testable alternative in addition to existing hypotheses, our model allows a more comprehensive investigation of both the proximate function and the evolution of genomic imprinting.

## Materials and Methods

### Derivation of maternal–offspring genotype frequencies for the single-locus model.

For the single-locus model, the frequencies of the 16 possible maternal–offspring genotype combinations at the *A* locus were derived under the assumption of a standard random mating population [51]. As a result, genotype frequencies in both mothers and their offspring conform to Hardy-Weinberg proportions. Furthermore, we assume that frequencies of the alleles are equal in the two generations, because they are measured at the same point in time (after selection in the maternal generation and before selection in the offspring generation). The maternal–offspring genotype frequencies are given in Table 1.

**Table 1:**
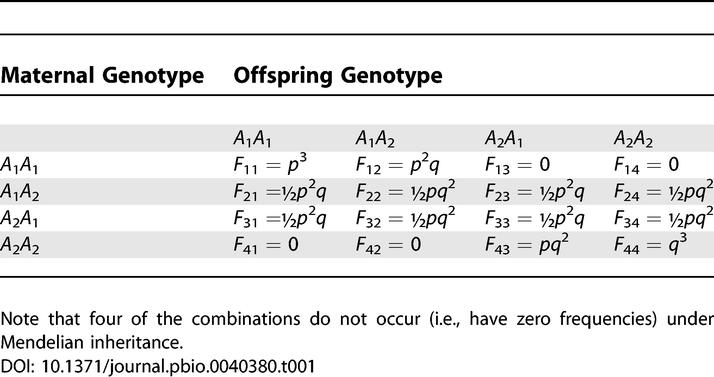
Frequencies of Maternal-Offspring Genotype Combinations for the Single-Locus Model

### Derivation of maternal–offspring genotype frequencies for the two-locus model.

We start by deriving the frequencies of maternal–offspring two-locus genotype combinations with the ultimate goal of deriving the frequencies of maternal *M*-locus–offspring *O*-locus genotype combinations. These frequencies are important because they are the unit to which fitness is assigned. See also [30] for a discussion of the structure and further details of this model.

The frequencies of the four possible two-locus haplotypes in the parental generation measured after selection but before gamete production [30] in that generation are given as: 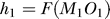 *,* 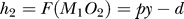 *,* 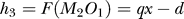 , and 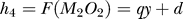 *,* where *d* is a measure of linkage disequilibrium [52]. The value of *d* in the offspring generation is equal to the value in the parental generation minus the amount lost due to recombination, such that *d*_offspring_ = *d*_parents_ – *rd*_parents_ *,* where *r* is the recombination rate between these two loci. Therefore, we use only one parameter, *d,* to measure linkage disequilibrium [30].

The 16 possible two-locus maternal genotypes are constructed from the products of the haplotype frequencies by factorial combination of the four haplotypes *M*_1_ *O*_1_ *, M*_1_ *O*_2_ *, M*_2_ *O*_1_ *,* and *M*_2_ *O*_2_. Although we assume that the maternal trait is not imprinted, we track parent-of-origin of alleles in mothers for simplicity, because we need to differentiate the parent-of-origin of alleles in offspring. The frequencies of the 16 maternal genotypes are denoted ϕ*_ij _,* where *i* and *j* correspond to the two haplotypes (*i* and *j* can be: 1 = *M*_1_ *O*_1_ *,* 2 = *M*_1_ *O*_2_ *,* 3 = *M*_2_ *O*_1_ *,* and 4 = *M*_2_ *O*_2_) from which the genotypes are derived. The values ϕ*_ij_* are calculated as the products of the frequencies of the two haplotypes that make up each particular genotype (e.g., ϕ_11_ is the frequency *M*_1_ *O*_1_ */ M*_1_ *O*_1_ mothers and has the value *h*_1_ *h*_1_). The frequencies of the paternal gamete types, denoted *γ_i_* with subscripts following *h_i_* above, have the values of *h_i_* minus the proportion of linkage disequilibrium lost to recombination during gametogenesis in males (i.e.,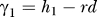 *,* 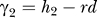 *,* 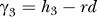 *,* and 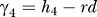 ).

The frequencies of the 256 possible maternal–offspring genotype combinations are denoted *C_ijkl_ ,* whereby the four subscripts define the two-locus genotypes of the mother and her offspring. The first two subscripts denote the mother's maternally and paternally inherited haplotypes, respectively, whereas the third and fourth subscripts denote the offspring's maternally and paternally derived haplotype respectively. The subscript numbering scheme follows that presented above for the haplotype frequencies. For example, the frequency of an *M*_1_ *O*_1_ */ M*_2_ *O*_2_ mother with an *M*_1_ *O*_1_ */ M*_1_ *O*_1_ offspring is *C_1411_*. These frequencies are a function of the following: (a) the frequencies of the 16 maternal genotypes, ϕ*_ij_ ,* (b) the frequencies of the paternal gametes, *γ _i_ ,* and (c) the rate of recombination, *r*. These frequencies are given in Table S1. Note that 144 of the 256 possible maternal–offspring genotype combinations do not exist under Mendelian inheritance.

These two-locus genotype frequencies can be used to derive the frequencies of the maternal *M*-locus–offspring *O*-locus combinations to which we assign fitness. These maternal–offspring genotype frequencies are denoted *F_ij_* **,** with subscripts following *w_ij_* (given in the Description of the Model section above) and are presented in Table 2. These frequencies are not two-locus genotype frequencies, but rather, are frequencies at which the various *O* locus genotypes in offspring are associated with the various *M* locus genotypes in their mothers.

**Table 2:**
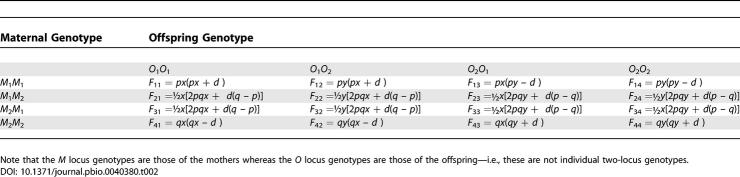
Frequencies of Maternal–Offspring Genotype Combinations for the Two-Locus Model

## Supporting Information

Table S1 Frequencies of Maternal–Offspring Genotype Combinations *(C_ijkl_)*
Maternal and offspring genotypes are given by their maternal and paternal haplotypes (listed as maternal/paternal). Empty cells have zero frequencies under Mendelian inheritance.
(112 KB DOC)
