# Supplementary material for: A Maternal–Offspring Coadaptation Theory for the Evolution of Genomic Imprinting
Source: PLoS Biol. 2006 Nov 14;4(12):e380. doi: 10.1371/journal.pbio.0040380 (PMC1635750; doi:10.1371/journal.pbio.0040380)
Supplement: Supplementary file 1 [file pbio.0040380.st001.doc]

**Table S1**. Frequencies of maternal-offspring genotype combinations (*C_ijkl_*). Maternal and offspring genotypes are given by their maternal and paternal haplotypes (listed as maternal/paternal). Empty cells have zero frequencies under Mendelian inheritance.

|  | Offspring genotypes | | | | | | | | | | | | | | | |
| --- | --- | --- | --- | --- | --- | --- | --- | --- | --- | --- | --- | --- | --- | --- | --- | --- |
| Maternal genotypes | *M_1_O_1_*/ *M_1_O_1_* | *M_1_O_1_*/ *M_1_O_2_* | *M_1_O_1_*/ *M_2_O_1_* | *M_1_O_1_*/ *M_2_O_2_* | *M_1_O_2_*/ *M_1_O_1_* | *M_1_O_2_*/ *M_1_O_2_* | *M_1_O_2_*/ *M_2_O_1_* | *M_1_O_2_*/ *M_2_O_2_* | *M_2_O_1_*/ *M_1_O_1_* | *M_2_O_1_*/ *M_1_O_2_* | *M_2_O_1_*/ *M_2_O_1_* | *M_2_O_1_*/ *M_2_O_2_* | *M_2_O_2_*/  *M_1_O_1_* | *M_2_O_2_*/ *M_1_O_2_* | *M_2_O_2_*/ *M_2_O_1_* | *M_2_O_2_*/ *M_2_O_2_* |
| *M_1_O_1_*/*M_1_O_1_* | *C*_1111_ =  *φ*_11_*γ*_1_ | *C*_1112_ =  *φ* _11_*γ*_2_ | *C*_1113_ =  *φ* _11_*γ*_3_ | *C*_1114_ =  *φ* _11_*γ*_4_ |  |  |  |  |  |  |  |  |  |  |  |  |
| *M_1_O_1_*/*M_1_O_2_* | *C*_1211_ =  ½*φ* _12_*γ*_1_ | *C*_1212_ =  ½*φ* _12_*γ*_2_ | *C*_1213_ =  ½*φ* _12_*γ*_3_ | *C*_1214_ =  ½*φ* _12_*γ*_4_ | *C*_1221_ =  ½*φ* _12_*γ*_1_ | *C*_1222_ =  ½*φ* _12_*γ*_2_ | *C*_1223_ =  ½*φ* _12_*γ*_3_ | *C*_1224_ =  ½*φ* _12_*γ*_4_ |  |  |  |  |  |  |  |  |
| *M_1_O_1_*/*M_2_O_1_* | *C*_1311_ =  ½*φ* _13_*γ*_1_ | *C*_1312_ =  ½*φ* _13_*γ*_2_ | *C*_1313_ =  ½*φ* _13_*γ*_3_ | *C*_1314_ =  ½*φ* _13_*γ*_4_ |  |  |  |  | *C*_1331_ =  ½*φ* _13_*γ*_1_ | *C*_1332_ =  ½*φ* _13_*γ*_2_ | *C*_1333_ =  ½*φ* _13_*γ*_3_ | *C*_1334_ =  ½*φ* _13_*γ*_4_ |  |  |  |  |
| *M_1_O_1_*/*M_2_O_2_* | *C*_1411_ =  ½*φ* _14_*γ*_1_  (1 – *r*) | *C*_1412_ =  ½*φ* _14_*γ*_2_  (1 – *r*) | *C*_1413_ =  ½*φ* _14_*γ*_3_  (1 – *r*) | *C*_1414_ =  ½*φ* _14_*γ*_4_  (1 – *r*) | *C*_1421_ =  ½*φ* _14_*γ*_1_*r* | *C*_1422_ =  ½*φ* _14_*γ*_2_*r* | *C*_1423_ =  ½*φ* _14_*γ*_3_*r* | *C*_1424_ =  ½*φ* _14_*γ*_4_*r* | *C*_1431_ =  ½*φ* _14_*γ*_1_*r* | *C*_1432_ =  ½*φ* _14_*γ*_2_*r* | *C*_1433_ =  ½*φ* _14_*γ*_3_*r* | *C*_1434_ =  ½*φ* _14_*γ*_4_*r* | *C*_1441_ =  ½*φ* _14_*γ*_1_  (1 – *r*) | *C*_1442_ =  ½*φ* _14_*γ*_2_  (1 – *r*) | *C*_1443_ =  ½*φ* _14_*γ*_3_  (1 – *r*) | *C*_1444_ =  ½*φ* _14_*γ*_4_  (1 – *r*) |
| *M_1_O_2_*/*M_1_O_1_* | *C*_2111_ =  ½*φ* _21_*γ*_1_ | *C*_2112_ =  ½*φ* _21_*γ*_2_ | *C*_2113_ =  ½*φ* _21_*γ*_3_ | *C*_2114_ =  ½*φ* _21_*γ*_4_ | *C*_2121_ =  ½*φ* _21_*γ*_1_ | *C*_2122_ =  ½*φ* _21_*γ*_2_ | *C*_2123_ =  ½*φ* _21_*γ*_3_ | *C*_2124_ =  ½*φ* _21_*γ*_4_ |  |  |  |  |  |  |  |  |
| *M_1_O_2_*/*M_1_O_2_* |  |  |  |  | *C*_2221_ =  *φ* _22_*γ*_1_ | *C*_2222_ =  *φ* _22_*γ*_2_ | *C*_2223_ =  *φ* _22_*γ*_3_ | *C*_2224_ =  *φ* _22_*γ*_4_ |  |  |  |  |  |  |  |  |
| *M_1_O_2_*/*M_2_O_1_* | *C*_2311_ =  ½*φ* _23_*γ*_1_*r* | *C*_2312_ =  ½*φ* _23_*γ*_2_*r* | *C*_2313_ =  ½*φ* _23_*γ*_3_*r* | *C*_2314_ =  ½*φ* _23_*γ*_4_*r* | *C*_2321_ =  ½*φ* _23_*γ*_1_  (1 – *r*) | *C*_2322_ =  ½*φ* _23_*γ*_2_  (1 – *r*) | *C*_2323_ =  ½*φ* _23_*γ*_3_  (1 – *r*) | *C*_2324_ =  ½*φ* _23_*γ*_4_  (1 – *r*) | *C*_2331_ =  ½*φ* _23_*γ*_1_  (1 – *r*) | *C*_2332_ =  ½*φ* _23_*γ*_2_  (1 – *r*) | *C*_2333_ =  ½*φ* _23_*γ*_3_  (1 – *r*) | *C*_2334_ =  ½*φ* _23_*γ*_4_  (1 – *r*) | *C*_2341_ =  ½*φ* _23_*γ*_1_*r* | *C*_2342_ =  ½*φ* _23_*γ*_2_*r* | *C*_2343_ =  ½*φ* _23_*γ*_3_*r* | *C*_2344_ =  ½*φ* _23_*γ*_4_*r* |
| *M_1_O_2_*/*M_2_O_2_* |  |  |  |  | *C*_2421_ =  ½*φ* _24_*γ*_1_ | *C*_2422_ =  ½*φ* _24_*γ*_2_ | *C*_2423_ =  ½*φ* _24_*γ*_3_ | *C*_2424_ =  ½*φ* _24_*γ*_4_ |  |  |  |  | *C*_2441_ =  ½*φ* _24_*γ*_1_ | *C*_2442_ =  ½*φ* _24_*γ*_2_ | *C*_2443_ =  ½*φ* _24_*γ*_3_ | *C*_2444_ =  ½*φ* _24_*γ*_4_ |
| *M_2_O_1_*/*M_1_O_1_* | *C*_3111_ =  ½*φ* _31_*γ*_1_ | *C*_3112_ =  ½*φ* _31_*γ*_2_ | *C*_3113_ =  ½*φ* _31_*γ*_3_ | *C*_3114_ =  ½*φ* _31_*γ*_4_ |  |  |  |  | *C*_3131_ =  ½*φ* _31_*γ*_1_ | *C*_3132_ =  ½*φ* _31_*γ*_2_ | *C*_3133_ =  ½*φ* _31_*γ*_3_ | *C*_3134_ =  ½*φ* _31_*γ*_4_ |  |  |  |  |
| *M_2_O_1_*/*M_1_O_2_* | *C*_3211_ =  ½*φ* _32_*γ*_1_*r* | *C*_3212_ =  ½*φ* _32_*γ*_2_*r* | *C*_3213_ =  ½*φ* _32_*γ*_3_*r* | *C*_3214_ =  ½*φ* _32_*γ*_4_*r* | *C*_3221_ =  ½*φ* _32_*γ*_1_  (1 – *r*) | *C*_3222_ =  ½*φ* _32_*γ*_2_  (1 – *r*) | *C*_3223_ =  ½*φ* _32_*γ*_3_  (1 – *r*) | *C*_3224_ =  ½*φ* _32_*γ*_4_  (1 – *r*) | *C*_3231_ =  ½*φ* _32_*γ*_1_  (1 – *r*) | *C*_3232_ =  ½*φ* _32_*γ*_2_  (1 – *r*) | *C*_3233_ =  ½*φ* _32_*γ*_3_  (1 – *r*) | *C*_3234_ =  ½*φ* _32_*γ*_4_  (1 – *r*) | *C*_3241_ =  ½*φ* _32_*γ*_1_*r* | *C*_3242_ =  ½*φ* _32_*γ*_2_*r* | *C*_3243_ =  ½*φ* _32_*γ*_3_*r* | *C*_3244_ =  ½*φ* _32_*γ*_4_*r* |
| *M_2_O_1_*/*M_2_O_1_* |  |  |  |  |  |  |  |  | *C*_3331_ =  *φ* _33_*γ*_1_ | *C*_3332_ =  *φ* _33_*γ*_2_ | *C*_3333_ =  *φ* _33_*γ*_3_ | *C*_3334_ =  *φ* _33_*γ*_4_ |  |  |  |  |
| *M_2_O_1_*/*M_2_O_2_* |  |  |  |  |  |  |  |  | *C*_3431_ =  ½*φ* _34_*γ*_1_ | *C*_3432_ =  ½*φ* _34_*γ*_2_ | *C*_3433_ =  ½*φ* _34_*γ*_3_ | *C*_3434_ =  ½*φ* _34_*γ*_4_ | *C*_3441_ =  ½*φ* _34_*γ*_1_ | *C*_3442_ =  ½*φ* _34_*γ*_2_ | *C*_3443_ =  ½*φ* _34_*γ*_3_ | *C*_3444_ =  ½*φ* _34_*γ*_4_ |
| *M_2_O_2_*/*M_1_O_1_* | *C*_4111_ =  ½*φ* _41_*γ*_1_  (1 – *r*) | *C*_4112_ =  ½*φ* _41_*γ*_2_  (1 – *r*) | *C*_4113_ =  ½*φ* _41_*γ*_3_  (1 – *r*) | *C*_4114_ =  ½*φ* _41_*γ*_4_  (1 – *r*) | *C*_4121_ =  ½*φ* _41_*γ*_1_*r* | *C*_4122_ =  ½*φ* _41_*γ*_2_*r* | *C*_4123_ =  ½*φ* _41_*γ*_3_*r* | *C*_4124_ =  ½*φ*_41_*γ*_4_*r* | *C*_4131_ =  ½*φ* _41_*γ*_1_*r* | *C*_4132_ =  ½*φ* _41_*γ*_2_*r* | *C*_4133_ =  ½*φ* _41_*γ*_3_*r* | *C*_4134_ =  ½*φ* _41_*γ*_4_*r* | *C*_4141_ =  ½*φ* _41_*γ*_1_  (1 – *r*) | *C*_4142_ =  ½*φ* _41_*γ*_2_  (1 – *r*) | *C*_4143_ =  ½*φ* _41_*γ*_3_  (1 – *r*) | *C*_4144_ =  ½*φ* _41_*γ*_4_  (1 – *r*) |
| *M_2_O_2_*/*M_1_O_2_* |  |  |  |  | *C*_4221_ =  ½*φ* _42_*γ*_1_ | *C*_4222_ =  ½*φ* _42_*γ*_2_ | *C*_4223_ =  ½*φ* _42_*γ*_3_ | *C*_4224_ =  ½*φ* _42_*γ*_4_ |  |  |  |  | *C*_4241_ =  ½*m*_42_*γ*_1_ | *C*_4242_ =  ½*m*_42_*γ*_2_ | *C*_4243_ =  ½*m*_42_*γ*_3_ | *C*_4244_ =  ½*m*_42_*γ*_4_ |
| *M_2_O_2_*/*M_2_O_1_* |  |  |  |  |  |  |  |  | *C*_4331_ =  ½*φ* _43_*γ*_1_ | *C*_4332_ =  ½*φ* _43_*γ*_2_ | *C*_4333_ =  ½*φ* _43_*γ*_3_ | *C*_4334_ =  ½*φ* _43_*γ*_4_ | *C*_4341_ =  ½*φ* _43_*γ*_1_ | *C*_4342_ =  ½*φ* _43_*γ*_2_ | *C*_4343_ =  ½*φ* _43_*γ*_3_ | *C*_4344_ =  ½*φ* _43_*γ*_4_ |
| *M_2_O_2_*/*M_2_O_2_* |  |  |  |  |  |  |  |  |  |  |  |  | *C*_4441_ =  *φ* _44_*γ*_1_ | *C*_4442_ =  *φ* _44_*γ*_2_ | *C*_4443_ =  *φ* _44_*γ*_3_ | *C*_4444_ =  *φ* _44_*γ*_4_ |
